# 

**Published:** 1879-08

**Authors:** 


### Table of Contents.

### ORIGINAL COMMUNICATIONS.

Rubeola and its Treatment. By E. A. Cobleigh, M.D., Athens, Tennessee........ 257
A Case of Constant and Protracted Vomiting from Pregnancy. By H. L. Wilson, M.D., Atlanta, Ga........ 265

### REPORTS OF SOCIETIES.

The Medical Society of the County of New York........ 267
New York Patholgoical Society........ 280

### SELECTIONS.

Physiological Antagonism the Therapeutic Law of Cure........ 284
Physiology of the Secretion of Sweat........ 291
Theories of Diseases—Preliminary Remarks........ 293

### EDITORIAL.

Yellow Fever not Personally Communicable........ 300
Meteorological Report for July, 1879........ 302
Editorial Correspondence........ 303

### BIBLIOGRAPHICAL.

Pocket Therapeutics and Dose Book, with Classification and Explanation of the Actions of Medicines........ 307
Manual of the Principles and Practice of Operative Surgery........ 307
Lessons in Gynæcology........ 308
A Clinical Treatise on the Diseases of the Nervous System........ 309
A Text Book of Physiology........ 309

### EXCERPTA.

The Healing of Arteries........ 311
Nitrite of Amyl in Chloral-Poisoning........ 313
Treatment of Colic........ 315
Use of Pilocarpinum Muriaticum in Children's Diseases........ 316
Contributions to the Pathological Anatomy of Acute Delirium........ 318
Poisoning........ 319
Plugging the Nasal Cavities........ 320
A Medical Contract in Italy........ 320
A Summer Madrigal........ 320

